# Professor Li-Pin. King: a famous physiologist in China

**DOI:** 10.1007/s13238-018-0582-z

**Published:** 2018-10-25

**Authors:** Wei Gong, Fangfang Wang, Yike Ying

**Affiliations:** Zhejiang Normal University, Jinhua, 321004 China

Professor Li-Pin King (经利彬, 1895–1958) is a famous physiologist in China. He devoted his entire life to the researches on Pharmacology of traditional Chinese medicine, Experimental Biology and Modern medicine. He made tremendous contributions to the development of Physiology in China. Prof. King is also the first scientist who applied the scientific methods to researches on traditional Chinese medicine and one of the earliest generations of Chinese researchers who studied modern medical science (Luo, 1993).

**Figure 1:**
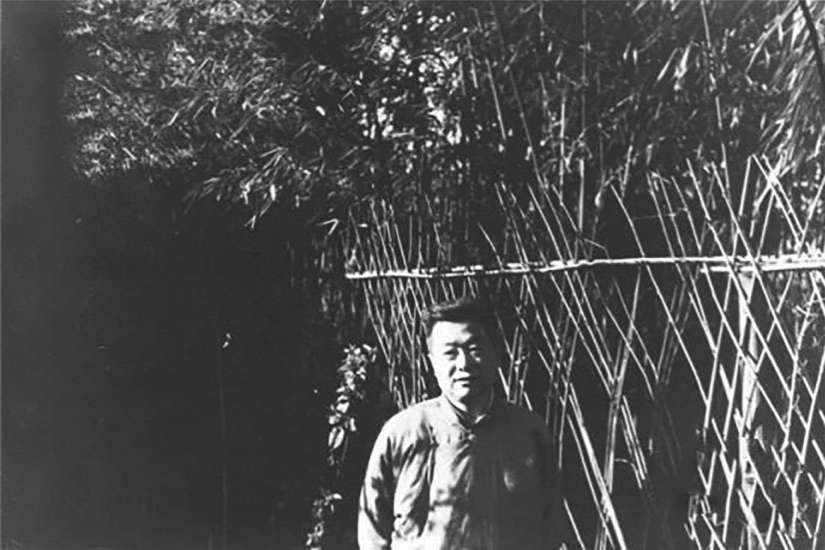
Professor Li-Pin King in Yunnan, China, 1943

In 1895, Prof. King was born in Zhejiang, China. He studied abroad at the University of Lyon in France and graduated with degrees in both science and medicine. After graduation, he served as a teaching assistant there. Later on, he returned back to China and became a professor at the Agricultural College of Peiping University and also was appointed as the director of the Department of Biology. In October 1929, Institute of Biology, National Academy of Peiping (later renamed as Institute of Physiology, National Academy of Peiping) was established and Prof. King was appointed as the director. He was also a professor of Peiping University, Peiping Women’s College of Arts and Sciences and Sun Yat-sen University. Unfortunately, Peiping was occupied by the invaders in 1937. Prof. King moved to Kunming, Yunnan together with the National Academy of Peiping and later served as professor in biology at Yunnan University and the director of the Chinese Medical Academy (Fig. 1). In 1946, Prof. King moved to Taiwan and was appointed as the director for the Health Bureau. But he resigned soon since he was not good at the official communications. Prof. King passed away in Taiwan in 1958.

Prof. King carried out researches on Nutrition, Pharmacology of traditional Chinese medicine, Experimental biology and Modern medicine, and published many research articles and monographs. According to *The Catalogue of the Publications of the National Academy of Peiping* published by the General Office of National Academy of Peiping in 1948, the number of articles published by the Institute of Physiology led by Prof. King was in the third place among ten research institutes, and the number of articles published in foreign journals ranked in the second place. All those publications significantly promoted the development of Physiology in China (Zhang, 2014).

Prof. King concerned about the health and nutrition of Chinese, therefore he focused on the researches on the relationship between food nutrients and nutritious elements essential for Chinese. For example, he studied food nutrients in northern China and its effects on the levels of phosphorus, calcium in human blood, showing the effects of ordinary food on people’s growth and health, and emphasizing the importance of diet. Prof. King also performed pharmacological researches on Chinese medicine systematically. His researches clarified the physiological effects of some Chinese herbs and their effects on blood components, blood pressure and organs in human (King and Shi, 1934), (King, 1935) which opened the way of using scientific research methods in Pharmacology to study traditional Chinese medicine. He also published his research data in an international journal *Biology* (Zhang, 2014), introducing the scientific principles of traditional Chinese medicine to benefit the foreigners. Prof. King was also interested in Experimental Biology. He collaborated with the aquatic animal research team in the Institute of Zoology and conducted experimental biological researches about water organisms (Academy of Peiping, 1936), which significantly promoted the development of Experimental Biology in China. Prof. King was also one of the earliest scientists who engaged in the studies of modern medical research in China. He studied and introduced systematical knowledge of serum, red blood cells and blood clots. In addition, he combined experimental biology with pharmacology of traditional Chinese medicines, and studied the effects of Chinese medicines and anesthetics on blood components, blood pressure etc., and published his research data on *Science*, one of the most influential scientific journals.

At the time when Prof. King was appointed as the director of the Institute of Physiology, National Academy of Peiping, there were only 4 researchers in the institute. He thus began to recruit and cultivate researchers and had a scientific research team of 17 persons, including two full-time researchers, a special researcher, a correspondent in Europe and several assistants (Zhang, 2014), which laid great foundation for further researches. Prof. King paid great attention to the collaborations between team members. Most of his researches were performed together with his team members, such as Yuangao Shi (石原皋), Yuqing Hou (侯玉清) and Yuhuang Zhao (赵燏黄), which benefited greatly the development of young scholars.

**Figure 2:**
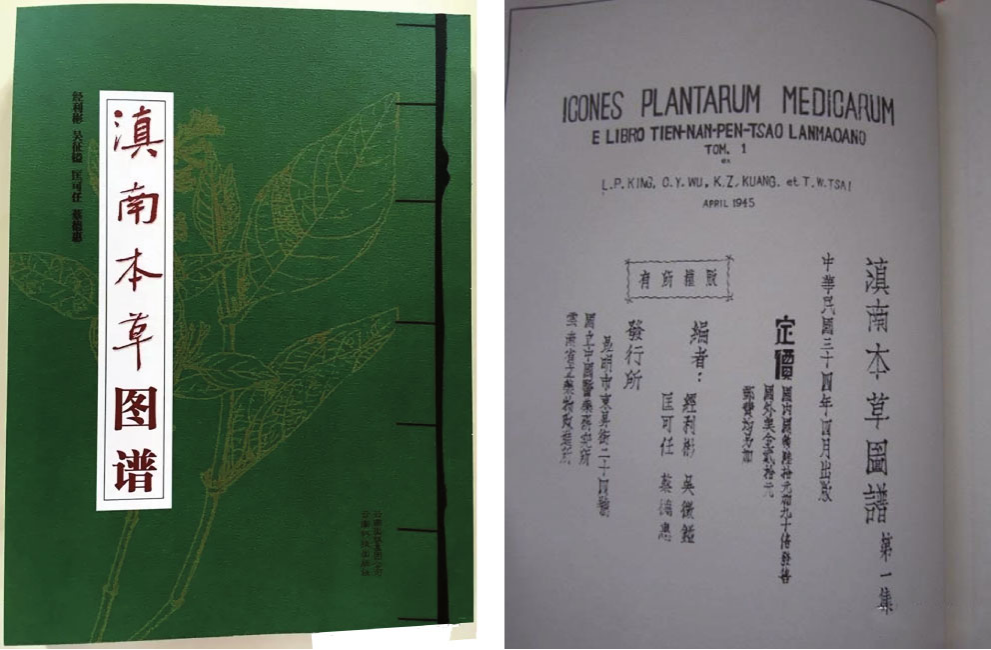
Icones Plantarum Medicarum (a photocopy in 2007)

Prof. King engaged in physiological researches all his life and compiled many books such as *The Brain Volume of Vertebrates*, *The Resurrection of Goldfish Fins and Scales*, *The Physiological Role of Sophora fruit*, etc., which had great impacts on Physiology. Prof. King also completed the book *Icones Plantarum Medicarum* together with other researchers, which was not only an important monograph in revitalizing Chinese medicine industry, but also a representative book of plant researches. In the first chapter of *Icones Plantarum Medicarum*, Prof. King selected 26 kinds of drugs in *Materia Medica of South Yunnan*, and drew the shapes of original plants, wrote instructions about their names, original texts, forms, relative researches, distributions, pharmacology and illustrations (Zhang and Li, 2004). Because of its importance, it has been republished multiple times (Fig. 2). In addition to academic researches, Prof. King also compiled *General Biology* independently, a university textbook, and collaborated with Prof. Chunzhi Wei (魏春芝) to compile *Physiological Hygiene*, a junior high school textbook, boosting biology and physiology education in China.

Prof. King was not only an outstanding researcher, but also an excellent educator who has cultivated many young talents. In September 1925, the Department of Biology of Peiping University was established and Prof. King was recruited as a lecturer to teach Physiology and Experimentation for the first three students of the Department: Fengying Zhang (张风灜), Yuangao Shi (石原皋) and Jingsheng Hao (郝景盛). One of them, Yuangao Shi, joined the research team led by Prof. King to study the pharmacological effects of Chinese medicine and the food nutrition in northern china and later on became a famous medical scientist in China. In addition, to support the students’ studies, Prof. King donated the book *Mai Jing* (*Pulse Classic*) written by Shuhe Wang (王叔和) to the National Yunnan University during his stay in Yunnan.

Prof. King was also one of the founders of Chinese Zoological Society. Together with other 29 scientists including Profs. Ti-Chow Tung (童第周) and Tchou Su (朱洗), Prof. King signed to establish the Chinese Zoological Society. In August 1934, Chinese Zoological Society was officially established in Jiangxi province, aiming to promote the development of Zoology in China. Prof. King was the second director and the fourth vice president of the society. Two years later, Profs. King, Tsung-Lê Loo (罗宗洛), Shitsan Pai (贝时璋) and Ti-Chow Tung set up *Chinese Journal of Experimental Biology* (later named as *Acta Biologiae Experimentalis Sinica*, now known as *Journal of Molecular Cell Biology*), which still has international influences and advances the development of Experimental Biology in China.

Prof. Li-Pin King is a great physiologist and made outstanding contributions to the development of physiology in China in terms of academic researches, cultivation of young talents, establishment of academic journals and research groups, especially in the areas of Chinese medicine pharmacology, experimental biology and modern medicine, etc. His rigorous research style, cooperative research spirit and collaborating research personality are worthy of appreciation and learning by scientific researchers.
